# Narcissism and Entrepreneurship: A Systematic Review and an Agenda for Future Research

**DOI:** 10.3389/fpsyg.2021.657681

**Published:** 2021-04-21

**Authors:** Dege Liu, Ting Zhu, Xiaojun Huang, Mansi Wang, Man Huang

**Affiliations:** ^1^School of Management (School of Tourism/Sino-French College of Tourism), Guangzhou University, Guangzhou, China; ^2^School of Innovation and Entrepreneurship, Guangzhou University, Guangzhou, China

**Keywords:** narcissism, personality, entrepreneurship, entrepreneurship theory, entrepreneurial process

## Abstract

Although narcissism is an important factor influencing entrepreneurial activity and outcomes, not much research has been conducted on the relationship between narcissism and entrepreneurship. To summarize the current literature on this relationship and provide an agenda for further in-depth research, a systematic review was conducted based on the PRISMA guidelines using Web of Science, Elsevier ScienceDirect, and EBSCO host databases. Accordingly, 33 articles have been identified as being eligible for the final synthesis. The findings of the present study showed, in general, that (1) life history theory, person-environment fit theory (P-E theory), and career choice theory were mostly used to explore the topic of narcissism and entrepreneurial intention, social exchange theory was used to analyze narcissistic entrepreneurs' entrepreneurial motives and attitudes, and upper echelons theory (UET) was applied to research on the relationship between narcissism and entrepreneurial outcomes, (2) Narcissistic Personality Inventory (NPI) and narcissistic sub-dimension of the Dark Triad were frequently used self-report scales among 23 identified empirical studies, and (3) narcissism has both bright and dark sides to entrepreneurial activities and outcomes. While narcissism makes potential entrepreneurs have higher entrepreneurial intentions and greater willingness to take risks, it also prevents entrepreneurs from discovering opportunities, acquiring resources, and learning from failure. Besides, results also showed that relations between narcissism and entrepreneurial intentions and performance are more complex. For a deeper understanding of this complex relations and advancing research on narcissism and entrepreneurship, more research is necessary to explore the relations between narcissism and entrepreneurship-related variables from a temporal perspective and at the team level, examine the relationship between narcissism and entrepreneurship ethics, and investigate the interaction effects of narcissism and other personalities.

## Introduction

In recent years, entrepreneurship has been a topic of concern for practitioners and researchers. Previous research yielded significant findings on entrepreneurial skills; knowledge and abilities; entrepreneurs' Big Five personality traits; as well as entrepreneurial cognition, emotion, attitude, identity, environment, and culture (Legge and Hindle, 2004; Miller, 2015; Omorede et al., 2015; Newman et al., 2019). However, despite the increasing popularity and significance of narcissism in organizational research (e.g., Grijalva and Harms, 2014; Grijalva et al., 2015), few studies have been conducted on the relationship between narcissism and entrepreneurship (Baldegger et al., 2017). Existing research confirms that entrepreneurs and non-entrepreneurs have distinct personality traits, such as risk propensity and locus of control (e.g., Stewart et al., 1999; Stewart and Roth, 2001; Zhao and Seibert, 2006). It also demonstrates how personality trait variables—such as the Big Five personality traits and proactive personalities—affect entrepreneurial intentions and performance (e.g., Collins et al., 2004; Zhao and Seibert, 2006; Zhao et al., 2010; Brandstatter, 2011; Obschonka and Stuetzer, 2017; Obschonka et al., 2019). Notably, the relationship between narcissism and entrepreneurship did not attract researchers' attention until very recently. It was not until 2013 that a study about this relationship was published (e.g., Mathieu and St-Jean, 2013). Omorede et al. (2015) found that entrepreneurial personality dominated early entrepreneurship research, from the 1960s to the early 2010s, with a significant spike in interest between 2005 and 2011. However, despite this demonstrated interest, the scarce research on the relationship between narcissism and entrepreneurship failed to reflect the critical impact that narcissism has on the entrepreneurial process and activities (Grijalva and Harms, 2014; Navis and Ozbek, 2016; Leung et al., 2021). This finding attests to the insufficient research on the relationship between narcissism and entrepreneurship, justifying our further investigation.

Entrepreneurship is a process wherein entrepreneurs identify, evaluate, and exploit entrepreneurial opportunities (Pryor et al., 2016). Preliminary research has confirmed that narcissism, wildly defined as a relatively stable personality construct consisting of grandiosity, self-love, inflated self-views, perception of entitlement, and preoccupation with success and achievement (Chatterjee and Hambrick, 2007; Campbell et al., 2011; Navis and Ozbek, 2016; Liu et al., 2019), has an impact on each stage of the entrepreneurial process and offers cognitional, motivational, and behavioral explanations for entrepreneurial activities and outcomes. For example, a study by Hmieleski and Lerner (2016) shows that narcissism is a crucial driver of entrepreneurial intentions, and is positively associated with productive and unproductive entrepreneurial motives. Bollaert et al. (2019) examined the effect of narcissism on crowdfunding, and found that narcissism affects the design of both the fundraising goal and campaign duration, as well as the success of the crowdfunding campaign. A research by Tucker et al. (2017) suggests that the sense of superiority, along with self-concern, self-protection, desire for admiration and recognition, and inherent self-improvement, prevent narcissistic individuals from effective use of resources. Such resources include advice and feedback from others during the discovery stage of entrepreneurial opportunities. Moreover, narcissists are more inclined to focus on entrepreneurial opportunities that will elicit praise and admiration, as opposed to evaluating opportunities based on their likelihood of success (Navis and Ozbek, 2016). They are also more aggressive in exploiting entrepreneurial opportunities, without depending on the abilities of others (e.g., utilizing competitive strategies).

Although previous studies have yielded some promising results, while revealing that narcissism may have significant positive and negative effects on entrepreneurship, they pay little attention to the mechanisms underscoring the relationship between narcissism and entrepreneurship, and thus leading to a paucity of studies on this relationship. It is unclear that (1) when and how narcissism affects entrepreneurial activities and processes, (2) what is the nature of the relationship between narcissism and entrepreneurial activities and their outcomes, (3) what level of narcissism is beneficial, and how to constructively stimulate its positive effects, and (4) how narcissistic entrepreneurs affect entrepreneurial team processes. Moreover, previous studies used different methodologies and tools to examine the relationship between narcissism and entrepreneurship, the integrative research that advances research on this relationship is still lacking. Therefore, a comprehensive, in-depth review, and analysis is needed to facilitate the development of robust research on the relationship between narcissism and entrepreneurship.

By systematically reviewing the literature on narcissism and entrepreneurship, the present study makes a number of contributions to the field. First, it lays the foundation for researchers to study the relations between narcissism and entrepreneurship in greater depth, thereby advancing the development of integral research on personality and entrepreneurship. In recent years, the relationship between personality and entrepreneurship has received greater interest from management and psychology researchers (e.g., Miller, 2015; Klotz and Neubaum, 2016). These studies suggest that personality traits can influence one or more aspects of the entrepreneurial process (e.g., Zhao et al., 2010; Murnieks et al., 2014; Obschonka and Stuetzer, 2017). Given the impact of narcissism on the entrepreneurial process and entrepreneurial activities, it should not be overlooked by researchers studying personality and entrepreneurship. Through in-depth analysis of previous research, this paper provides a comprehensive understanding of the key entrepreneurship variables affected by narcissism, how and why these variables are affected, when narcissism has a positive or negative effect on them, and how contextual factors affect the relationship between narcissism and these variables. Addressing these points lays a foundation for further investigation while enhancing the understanding of the relationship in question.

Second, the present study expands our understanding of the positive aspects of dark personalities. Because evidence links narcissism to self-interest, self-centeredness, emotional coldness, callousness, insensitivity, duplicity, exploitation, aggression, and deceptive tactics in previous studies, researchers largely consider it a dark personality trait (Paulhus and Williams, 2002; Jones and Paulhus, 2017). Indeed, it can cause many negative effects, ultimately bringing harm to individuals and business organizations. For example, narcissism can increase an individual's counterproductive behavior, create interpersonal barriers, and lead to ineffective management (O'Boyle et al., 2012; Spain et al., 2014; Smith et al., 2018).

Contrary to this perspective, however, recent research demonstrates that narcissism does not always have negative effects. It may enable individuals to achieve their desired positions (e.g., leadership positions, Campbell et al., 2011), boost their entrepreneurial self-efficacy and intentions (e.g., Mathieu and St-Jean, 2013; Wu et al., 2019b), and help them identify entrepreneurial opportunities (e.g., Tucker et al., 2016). By systematically collating studies concerning the relationship between narcissism and entrepreneurship, this study presents researchers with a more thorough understanding of the positive and negative effects the narcissistic personality has on entrepreneurial activities and outcomes. Moreover, this study will also enhance our understanding of the positive aspects of dark personalities, while responding to the call for more research in this area (e.g., Spain et al., 2014). This will, in turn, lay a foundation for future researchers to move beyond the traditional, binary paradigm that defines light as good and dark as bad.

Third, and most importantly, this study makes a critical contribution by identifying neglected research fields that require further investigation. To the best of our knowledge, this study is the first to systematically analyze the relationship between narcissism and entrepreneurship. By presenting an in-depth analysis of the existing research on that relationship, we uncover neglected research areas for future researchers, identify unresolved issues that need further attention, highlight possible future research opportunities, and determine the main avenues and directions for future research. Accordingly, this study contributes to the further development of this field.

In terms of structure, the remaining portion of present study begins with a concise description of the methodology used to research the literature related to this topic before conducting an in-depth and systematic analysis. And then, we analyze the findings of previous studies with respect to the theoretical foundations of research on narcissism and entrepreneurship, the measurement of narcissism, and the relationship between narcissism and entrepreneurship. Finally, based on the existing literature and our in-depth understanding, we identify critical research directions and opportunities requiring the attention of future researchers.

## Methods

**Figure 1:**
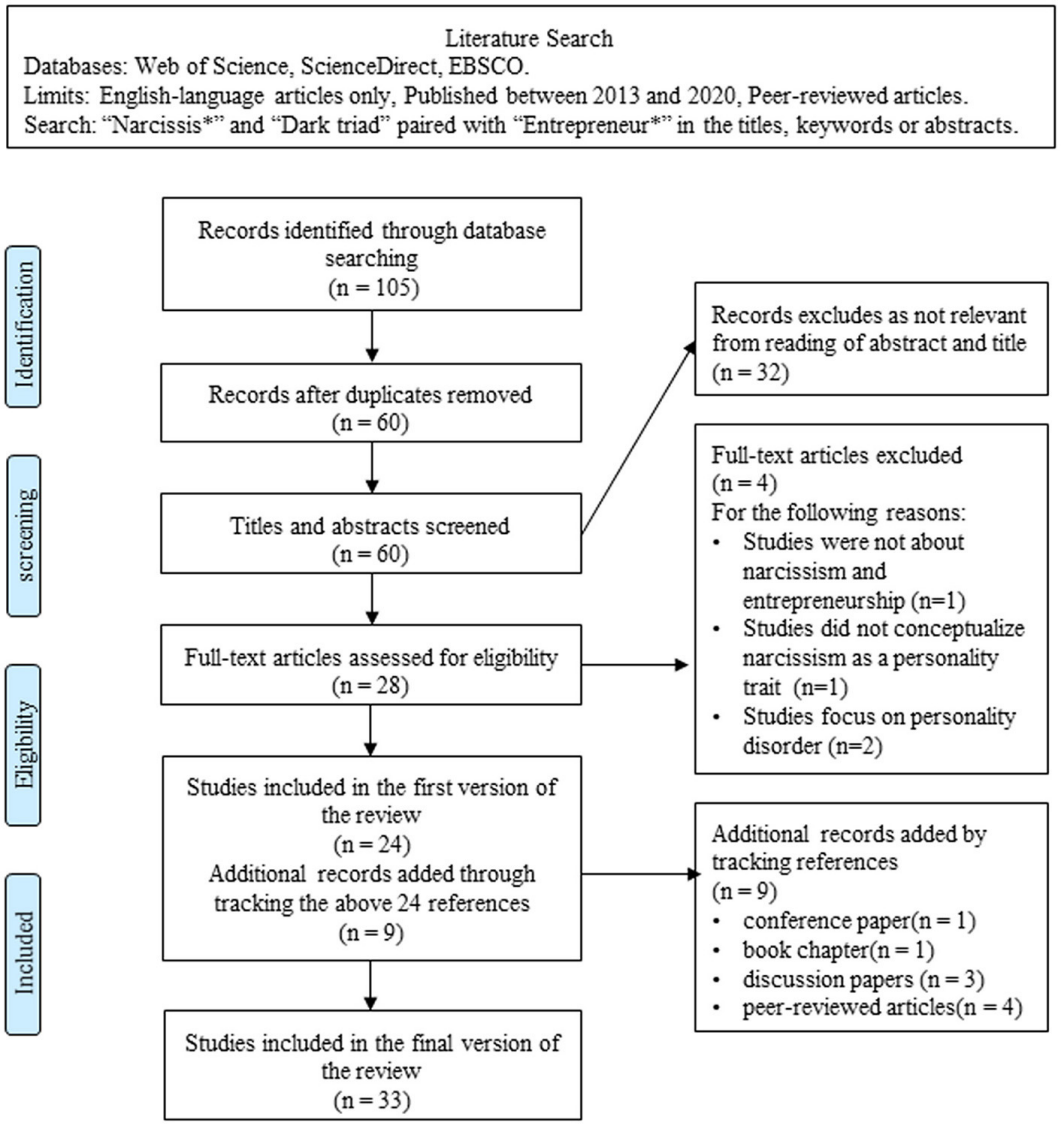
PRISMA flow diagram.

The systematic review was conducted following the guidelines for systematic reviews and meta-analysis (PRISMA, Moher et al., 2009). A flow diagram of the search process is presented in Figure 1. Between August 2019 and December 2020, we used the Web of Science, Elsevier ScienceDirect and EBSCO (PsycINFO and PsycARTICLE) databases to identify peer-reviewed articles with “narcissis^*^” and “dark triad” paired with “entrepreneur^*^” in their titles, keywords, or abstracts. Considering it wasn't until 2013 that the first study focusing on narcissism and entrepreneurship was published (e.g., Mathieu and St-Jean, 2013), we limited our search to those published between the beginning of 2013 and the end of 2020 and written in English. The preliminary search yielded 105 articles. After excluding duplicates, 60 articles were remained.

To select primary studies for systematic review, the first two authors read the titles, keywords, and abstracts of these articles simultaneously and independently, and cross-checked whether the article is related to narcissism and entrepreneurship, we only included those related to them. After the preliminary selection, 28 articles remained.

Then, the last three authors simultaneously and independently read the full text of the 28 articles, evaluated, cross-checked, and excluded studies that clearly did not meet the following criteria. First, the study must conceptualize narcissism as a personality trait that exists along a continuum from low to high levels (Campbell and Campbell, 2009; Campbell et al., 2011). Second, the study must not focus on personality disorder (e.g., pathological narcissism or narcissistic personality disorder), as pathological narcissism is an extreme and rare case that elicits qualitatively different behaviors to grandiose narcissism (Navis and Ozbek, 2016). Besides, we excluded one study that we could not clearly determine whether the article was relevant to the topic by reading the title, abstract, and key words (e.g., Upsides to dark and downsides to bright personality: a multidomain review and future research agenda, Smith et al., 2018). As a result, 24 articles were identified as closely related to the topic we sought to explore.

To identify more literature on this relationship, and ensure a comprehensive review of the literature, we used several other approaches to supplement our search. First, we checked the references of all currently included studies to identify additional articles of interest. Second, we surveyed previous reviews (e.g., Frese and Gielnik, 2014; Omorede et al., 2015) and meta-analyses (e.g., Zhao and Seibert, 2006; Zhao et al., 2010) related to entrepreneurship psychology and personality to find relevant studies. We thus obtained nine additional sources, which include one conference paper (e.g., Stöckmann et al., 2015), one book chapter (e.g., Tucker et al., 2016), three discussion papers (e.g., DeNisi, 2015; Miller, 2015; Klotz and Neubaum, 2016), and four peer-reviewed articles (Leonelli et al., 2016, 2019; Tucker et al., 2017; Kraus et al., 2018).

## Results and Findings

### Summary of the Results

Using the methods above, a total of 33 articles were found, of which, 23 were empirical studies, 5 conceptual research, 4 discussion papers, and 1 book chapter. Information on the author of the study, published year, journal, sample, measure of narcissism, and types of data was presented in Table 1. As is shown in Table 1, of the 23 empirical studies, most use cross-sectional design (65.22%) and self-reported data (86.96%) to conduct their research, only a few studies used secondary data (17.39%) and longitudinal study designs (17.39%). Narcissistic Personality Inventory (NPI, 8 articles) and narcissistic sub-dimension of the Dark Triad (7 articles) were mostly used scales to measure narcissism accounting for 65.22%. Table 2 presents research theme, primary research questions, main findings, and contributors. As can be seen, previous research used different theories to investigate six topics including entrepreneurial Intention, opportunity recognition, resource acquisition, risk-taking, learning from failure, and performance. In the following section, the findings are presented along theoretical foundation, definition, and measurement of narcissism and these six aspects.

**Table 1:** Overview of included papers.

**References**	**Journal**	**Method**	**Theory**	**Sample**	**Measure of narcissism**	**Research design**	**Types of data**
Ahsan, 2017	Academy of Management Review	Discussible	*Unstated*	–	–	–	–
Al-Ghazali and Afsar, 2020	Journal of High Technology Management Research	Empirical	Life history theory	362 Employees in technology incubators and science parks	8-Item scale, Resick et al., 2009	Cross-sectional design	Self-reported data
Baldegger et al., 2017	International Journal of Entrepreneurial Venturing	Empirical	Action-characteristics model of entrepreneurship	385 Students in business administration	NARQ, Back et al., 2013	Cross-sectional design	Self-reported data
Bollaert et al., 2019	Small Business Economics	Empirical	*Unstated*	14,968 Crowdfunding campaigns from Indiego	Personal pronouns, Raskin and Shaw, 1988	*Unstated*	Secondary data
Bouncken et al., 2020	International Journal of Entrepreneurial Venturing	Empirical	Upper echelon theory	191 Business-owners and top managers	4-Item sub-measure from Dirty Dozen, Jonason and Webster, 2010	Cross-sectional design	Self-reported data
Butticè and Rovelli, 2020	Personality and Individual Differences	Empirical	*Unstated*	59,538 Crowdfunding campaigns from Kickstarter	Personal pronouns, Raskin and Shaw, 1988	*Unstated*	Secondary data
DeNisi, 2015	Entrepreneurship Theory and Practice	Discussible	*Unstated*	–	–		–
Do and Dadvari, 2017	Asia Pacific Management Review	Empirical	Life history theory	295 Undergraduate students taking business administration	Nine items in the SD3, Jones and Paulhus, 2014	Cross-sectional design	Self-reported data
Engelen et al., 2016	Journal of Management	Empirical	Upper echelon theory	High-tech companies in USA	Proxy indicators, Chatterjee and Hambrick, 2007	Panel design	Secondary data
Grijalva and Harms, 2014	Academy of Management Perspectives	Conceptual	*Unstated*	–	–	–	–
Hmieleski and Lerner, 2016	Journal of Small Business Management	Empirical	Life history theory and social exchange theory	508 Business undergraduates and 234 MBA students	NPI-40, Raskin and Terry, 1988	Cross-sectional design	Self-reported data
Jackson, 2018	Personality and Individual Differences	Empirical	*Unstated*	227 Fulltime managers from the USA	Nine items in the SD3, Jones and Paulhus, 2014	Cross-sectional design	Self-reported data
Klotz and Neubaum, 2016	Entrepreneurship Theory and Practice	Conceptual	*Unstated*	–	–	–	–
Kollmann et al., 2019	Journal of Small Business Management	Empirical	*Unstated*	132 Team members	NPI-16, Ames et al., 2006	Longitudinal design	Self-reported data
Kraus et al., 2018	Journal of Promotion Management	Empirical	*Unstated*	131 CEOs and company owners	4-Item sub-measure from Dirty Dozen, Jonason and Webster, 2010	Cross-sectional design	Self-reported data
Leonelli et al., 2016	Sinergie Italian Journal of Management	Conceptual	*Unstated*	–	–	–	–
Leonelli et al., 2019	Sinergie Italian Journal of Management	Empirical	Upper echelon theory	115 Italian cross-industry entrepreneurs	NPI-16, Ames et al., 2006	Cross-sectional design	Self-reported data
Liu et al., 2019	Journal of Business Venturing	Empirical	*Unstated*	180 Founders of new ventures	NPI-16, Ames et al., 2006	Cross-sectional design	Self-reported data
Mathieu and St-Jean, 2013	Personality and Individual Differences	Empirical	Career choice theory and person-environment fit theory	108 entrepreneurial students;73 students;98 financial industry employees;116 city workers	NPI-16, Ames et al., 2006	Longitudinal design	Self-reported data
Miller, 2015	Entrepreneurship Theory and Practice	Conceptual	*Unstated*	–	–	–	–
Miller, 2016	Entrepreneurship Theory and Practice	Discussible	*Unstated*	–	–	–	–
Navis and Ozbek, 2016	Academy of Management Review	Conceptual	*Unstated*	–	–	–	–
Navis and Ozbek, 2017	Academy of Management Review	Discussible	*Unstated*	–	–	–	–
Presenza et al., 2020	Current Issues in Tourism	Empirical	*Unstated*	89 Members of the Italian Tourism Startups Association	*Unstated*	Cross-sectional design	Self-reported data
Shabbir and Kousar, 2019	Asia Pacific Journal of Innovation and Entrepreneurship	Empirical	Upper echelon theory	121 CEOs of private schools	NPI-16, Ames et al., 2006	Cross-sectional design	Self-reported data
Stöckmann et al., 2015	Academy of Management Proceedings	Empirical	Person–environment fit theory	66 Teams of two students	NPI-16, Ames et al., 2006	Longitudinal design	Self-reported data data
Tucker et al., 2016	Book chapter	Conceptual	*Unstated*	–	–	–	–
Tucker et al., 2017	Journal of Business and Entrepreneurship	Empirical	Expectancy theory	221 Working professionals	4-Item sub-measure from Dirty Dozen, Jonason and Webster, 2010	Longitudinal design	Self-reported data
Wales et al., 2013	Journal of Management Studies	Empirical	Upper-echelons theory	173 CEOs in high-tech manufacturing frims	NPI-16, Ames et al., 2006	*Unstated*	Self-reported and secondary data
Wu et al., 2019a	Frontiers in Psychology	Empirical	Social exchange theory and social cognition theory	334 MBA students	4-Item sub-measure from Dirty Dozen, Jonason and Webster, 2010	Cross-sectional design	Self-reported data
Wu et al., 2019b	Frontiers in Psychology	Empirical	Life history theory	334 MBA students	4-Item sub-measure from Dirty Dozen, Jonason and Webster, 2010	Cross-sectional design	Self-reported data
Wu et al., 2020	Frontiers in Psychology	Empirical	Social cognition theory	491 Students	4-Item sub-measure from Dirty Dozen, Jonason and Webster, 2010	Cross-sectional design	Self-reported data
Yu et al., 2020	Economic Research-Ekonomska IstraŽivanja	Empirical	Goal-setting theory and upper echelon theory	347 Entrepreneurial teams	8-Item scale, Resick et al., 2009	Cross-sectional design	Self-reported data

**Table 2:** Research themes, primary research questions, main findings, and contributors.

**Research theme**	**Primary research question**	**Main findings**	**Contributors**
Narcissism and entrepreneurial intention	Whether narcissists have higher entrepreneurial intentions and whether entrepreneurs are more narcissistic.	Narcissists have higher entrepreneurial intentions and entrepreneurs have higher levels of narcissism.	Mathieu and St-Jean, 2013
	The relationship between narcissism and entrepreneurial intention.	Narcissism positively affects entrepreneurial intention.	Mathieu and St-Jean, 2013; Hmieleski and Lerner, 2016; Do and Dadvari, 2017; Jackson, 2018; Wu et al., 2019a; Al-Ghazali and Afsar, 2020
		Narcissism positively affects intrapreneurship intention.	Tucker et al., 2017
		The relationship between narcissism and entrepreneurial intention is a U shape.	Wu et al., 2019b
		Narcissistic admiration positively predicts entrepreneurial intention while narcissistic rivalry negatively predicts entrepreneurial intention.	Baldegger et al., 2017
	What moderates the relationship between narcissism and entrepreneurial intention.	Resilience weakens the positive effects that narcissism has on entrepreneurial intention.	Wu et al., 2019a
		Entrepreneurial self-efficacy weakens the positive effects that narcissism has on intrapreneurship intention.	Tucker et al., 2017
	How narcissism affects entrepreneurial intention.	Narcissism influences entrepreneurial intention through entrepreneurial self-efficacy.	Wu et al., 2019b; Al-Ghazali and Afsar, 2020
		Dual narcissism influences entrepreneurial intention through career motivation.	Baldegger et al., 2017
Narcissism and opportunity recognition	The relationship between narcissism and opportunity recognition.	Narcissists are more inclined to focus on entrepreneurial opportunities that will elicit praise and admiration.	Navis and Ozbek, 2016, 2017; Tucker et al., 2016; Ahsan, 2017
Narcissism and resource acquisition	How narcissism affects resource acquisition.	Narcissistic individuals are more likely to acquire resources in the early stages of a relationship, but less likely to establish a long-term exchange of benefits.	Miller, 2015; Hmieleski and Lerner, 2016; Navis and Ozbek, 2016
		Narcissistic entrepreneurs were less likely succeed in crowdfunding.	Bollaert et al., 2019; Butticè and Rovelli, 2020
Narcissism and risk-taking	The relationship between narcissism and risk-taking propensity.	The radical, bold, decisive, and entirely self-confident nature of narcissists creates a greater willingness to take risks.	Grijalva and Harms, 2014; Navis and Ozbek, 2016
Narcissism and learning from failure	The relationship between narcissism and learning from failure.	Entrepreneurs' narcissism is not conducive to their learning from entrepreneurial failures.	Liu et al., 2019
Narcissism and entrepreneurial performance	The role of entrepreneurially-oriented strategy in the relationship between CEOs' narcissism and firm performance.	CEOs' narcissism positively affects entrepreneurially-oriented strategy, which leads to performance fluctuations.	Wales et al., 2013
		Narcissism weakens the positive effects that entrepreneurial orientation has on firm performance.	Engelen et al., 2016; Bouncken et al., 2020
		Executives' narcissism has no significant effect on the relationship between entrepreneurial orientation and performance.	Kraus et al., 2018
	How narcissism affects entrepreneurial performance.	The relationship between narcissism and entrepreneurial innovation is an inverted U-shape.	Leonelli et al., 2019
		Narcissism positively affects business plan performance by influencing entrepreneurial self-efficacy and entrepreneurial orientation.	Stöckmann et al., 2015

### Main Findings

#### Theoretical Foundations of Narcissism and Entrepreneurship Research

Previous studies used a variety of theories to investigate the topics of narcissism and entrepreneurship (see Tables 1, 2). To explore the topic of narcissism and entrepreneurial intention, the most common were life history theory (four articles, e.g., Hmieleski and Lerner, 2016; Do and Dadvari, 2017; Wu et al., 2019b; Al-Ghazali and Afsar, 2020), person-environment (P-E) fit theory (two articles, e.g., Mathieu and St-Jean, 2013; Stöckmann et al., 2015), and career choice theory (one article, e.g., Mathieu and St-Jean, 2013). These theories explained the effects of narcissism on entrepreneurial intention from the perspectives of cognition and motivation. The action-characteristics model of entrepreneurship was also used (e.g., Baldegger et al., 2017) to explain the relationship between narcissism and entrepreneurial intention. Notably, because action-characteristics model is a descriptive and loose model (Frese and Gielnik, 2014), we will not discuss it in detail in this section. Researchers have used social exchange theory to analyze narcissistic entrepreneurs' entrepreneurial motives and attitudes (two articles, e.g., Hmieleski and Lerner, 2016; Wu et al., 2019a). Research on the relationship between narcissism and entrepreneurial outcomes (e.g., entrepreneurial performance, success, and failure) mostly applies the upper echelons theory (UET; seven articles, e.g., Wales et al., 2013; Engelen et al., 2016; Kraus et al., 2018; Leonelli et al., 2019; Shabbir and Kousar, 2019; Bouncken et al., 2020; Yu et al., 2020). This is done to explore the impact of narcissism among entrepreneurs or senior management on corporate performance.

More specifically, life history theory points that individuals choose behavioral strategies (based on their environment) to maximize their likelihood of adaptation and survival, including growth, bodily maintenance, mating, and parenting (Buss, 2009; Del Giudice, 2014). In a threatening situation (e.g., a resource shortage) or highly uncertain environment, individuals tend to adopt a fast life strategy. This manifests as a preference for smaller, instant rewards, high-risk-taking behaviors, and short-term investments, short-term need fulfillment, and building short-term relationships. When the environment is less hostile and the future is predictable, individuals often adopt a slow life strategy, which manifests as a focus on long-term goals and investments, building long-term interpersonal relationships, and enhancing long-term survival (Kruger et al., 2008; Del Giudice, 2014).

Life history theory provides an extensive foundation based on which to examine the behavioral intentions and strategies of individuals in the entrepreneurial process. Accordingly, previous researchers have used it to explain the entrepreneurial intentions and motivations of narcissistic individuals. For example, Al-Ghazali and Afsar (2020), Wu et al. (2019b) and Hmieleski and Lerner (2016) believe that since highly narcissistic individuals perceive themselves as superior to, as well as smarter and more attractive than others, they constantly seek admiration and attention from others, as well as superiority and power, while taking risks to achieve greater benefits and achievements. To narcissists, entrepreneurship may be an effective way to meet these motivational needs. Thus, individuals with high levels of narcissism are more inclined to regulate themselves by adopting fast life strategies: by increasing their entrepreneurial intentions and choosing entrepreneurship, for instance.

According to P–E fit theory, P–E fit reflects the individual's degree of compatibility or fit with specific aspects of their work environment. Individuals are attracted to environments that are compatible with their attitudes, values, knowledge, skills, abilities, and personality. An environment that is good fit not only facilitates positive experiences and attitudes (e.g., more organization commitment, job satisfaction, and intent to stay), but also enables improved performance (Kristof-Brown et al., 2005; Oh et al., 2014). Moreover, P–E fit has stronger explanatory power for the individual's outcome than either personal or environmental factors alone. Conversely, P–E misfit will reduce positive outcomes, such as, decreasing satisfaction, and increasing psychological strain and turnover (Tanner et al., 2017; Bermiss and McDonald, 2018; Van Vianen, 2018). Similarly, Career choice theory also stipulates that the fit between an individual's values, personalities, and needs with the profession and work environment is a crucial factor influencing an individual's attitudes and wellbeing, and that individuals actively seek out occupations and workplaces that best match their values, needs and personality (Osipow, 1990).

The value of P–E fit theory and career choice theory are that they provide a theoretical basis for understanding individual emotions, attitudes, and behaviors. Researchers who focus on narcissism and entrepreneurship (e.g., Mathieu and St-Jean, 2013) have used them to explain the relationship between narcissism and entrepreneurial intentions. They argue that entrepreneurship is able to attract more social attention and satisfy narcissists' pursuit of status, reputation, power, and attention from others. Moreover, entrepreneurship maintains their sense of superiority, while also fitting the narcissist's risk propensity, inflated views of their entrepreneurial self-efficacy, and lack of fear of failure (Mathieu and St-Jean, 2013; Navis and Ozbek, 2016). Thus, narcissistic individuals will choose this route because entrepreneurship supports their values and characteristics.

Social exchange theory maintains that interactions between members of a society involve the exchange of various resources they valued (tangible or intangible) according to the reciprocity norm (Cropanzano and Mitchell, 2005; Blau, 2017). Individuals foster relationships and engage in reciprocal transactions with persons who can provide them with benefit return in the future. As the interaction evolves, a high-quality social relationship—characterized by mutual trust, loyalty, gratitude, commitment, and feelings of personal obligations—is formed between the actor and the target (Cropanzano and Mitchell, 2005).

Social exchange theory provides a valuable theoretical framework for understanding the resource acquisition, sustainable entrepreneurial orientation, and (un)productive entrepreneurial motives of narcissistic entrepreneurs (Hmieleski and Lerner, 2016; Wu et al., 2019a). For example, Hmieleski and Lerner's (2016) research indicates that narcissists are characterized by selfishness and dominance, and value short-term gratification. To maximize their interests, they avoid rewarding others for the provision of reciprocal benefits later on. This is done to attain resources and value rather than creating them (unproductive entrepreneurial motives). This echoes O'Boyle et al. (2012) who state that narcissists often display an aggressive and exploitative nature in their interpersonal relationships, to achieve personal goals. Grijalva and Harms (2014) also emphasized that narcissists' tendency to seek unilateral benefits undermines the reciprocal and interdependent relationship between the two parties, making narcissists unable to sustain long-term, cooperative partnerships.

Upper echelons theory points that the experiences, values, and personalities of top executives can influence the type and scope of the information they acquired, and determine how it is selected and interpreted (Hambrick and Mason, 1984; Hambrick, 2007). This, in turn, influences a company's strategic decisions and actions, as well as its performance (Hambrick and Mason, 1984; Hambrick, 2007). Upper echelons theory provides a formidable perspective from which to address the manner in which top executives influence the firm's strategic choices and performance. The importance of UET has been evident in many theoretical and empirical studies (see Wang et al., 2016). In the field of narcissism and entrepreneurship research, UET provides an essential theoretical foundation for understanding the mechanisms that underpin the effect of the chief executive officers' (CEOs) narcissism on firms' strategic decisions and performance. For example, based on this theory, Wales et al. (2013) confirmed that narcissistic CEOs are more likely to adopt entrepreneurially-oriented strategies, which, in turn, cause extreme variability in firm performance. Engelen et al.'s (2016) study showed that CEOs' narcissism tends to weaken the positive correlation between their firms' entrepreneurial orientation and performance.

#### Definition and Measurement of Narcissism in Entrepreneurship Research

The concept of “narcissism” can be traced back to the ancient Greek myth of “narcissus,” in which a man fell deeply in love with himself and succumbed to inextricable self-appreciation and adoration (Hermans and Van Gilst, 1991; Judge et al., 2006). Ellis (1898) introduced narcissism to the field of psychology to describe a pathological form of self-focus. As research evolved, the understanding of narcissism varied across disciplines. In clinical psychology and psychoanalysis, narcissism is considered a psychological (e.g., Ellis, 1898) or personality disorder (i.e., Narcissistic Personality Disorder, NPD for short, Campbell et al., 2011). Therein, it describes the individual's persistent and extreme self-love, self-inflation, pursuit of admiration, lack of empathy, pursuit of perfection, and inordinate sense of entitlement. In personality/social psychology and management, narcissism is conceptualized as a personality trait which share many similar characteristics with NPD (e.g., lack empathy, exploit others) and exists along a continuum: from low to high levels (Campbell and Campbell, 2009; Campbell et al., 2011). And it is deemed a common phenomenon that is found in all individuals with varying degrees (Raskin and Terry, 1988; Grijalva and Harms, 2014).

According to personality trait perspective, narcissistic individuals perceive themselves as superior to others, exhibit a grandiose view of themselves, have a sense of entitlement; self-love and self-inflation, pursue power, fame, and leadership positions, and show low levels of empathy and intimacy (Campbell and Campbell, 2009; Campbell et al., 2011). They have a higher need for sustained attention and admiration from others (Bogart et al., 2004; Wiklund et al., 2018), a stronger motivation for self-improvement, and more willingness to engage in self-improvement behaviors (Campbell et al., 2000). In the existing research on the relationship between narcissism and entrepreneurship, almost all researchers focused on narcissism as a personality trait, whether they considered it an independent concept or a dimension of the dark triad. They examined the impact of narcissistic personality on entrepreneurial activities and outcomes.

Concerning the measurement of narcissism in relation to entrepreneurship, previous studies predominantly used self-report scales (e.g., NPI-40, Raskin and Terry, 1988; NPI-16, Ames et al., 2006; and the 4-item sub-measure from Dirty Dozen scale, Jonason and Webster, 2010) and proxy indicators (e.g., Chatterjee and Hambrick, 2007). Table 1 lists the main instruments and methods used in narcissism and entrepreneurship research.

Of the extant measures, the NPI (Raskin and Hall, 1981) is the most frequently used self-reporting scale. In it, respondents' narcissism levels are assessed through their answers answer to 40 (NPI-40) or 16 (NPI-16) pairs of forced-choice questions. Among the 23 empirical studies included in this research, only 1 used the NPI-40 to measure entrepreneurs' narcissism with reference to 7 components: authority, superiority, self-sufficiency, entitlement, exploitative tendencies, exhibitionism, and vanity (e.g., Hmieleski and Lerner, 2016). However, many NPI-40 questions may induce time pressure and response fatigue. Thus, Ames et al. (2006) selected and validated 16 pairs of items from the NPI-40, thus forming the NPI-16, which correlated highly with the longer original scale. Moreover, this shorter scale has been widely used in the study of narcissism and entrepreneurship. Among the 23 empirical studies we retained, 7 adopted the NPI-16 to measure narcissism (e.g., Mathieu and St-Jean, 2013; Wales et al., 2013; Stöckmann et al., 2015; Kollmann et al., 2019; Leonelli et al., 2019; Liu et al., 2019; Shabbir and Kousar, 2019).

Entrepreneurial researchers have also used the subscales of dark traits to measure narcissism. For example, the Dirty Dozen scale, developed by Jonason and Webster (2010), has been used in several studies (e.g., Tucker et al., 2017; Kraus et al., 2018; Wu et al., 2019a,b, 2020; Bouncken et al., 2020). The Dirty Dozen scale consists of four narcissism-related items, such as the expectation of others' admiration or praise. Jones and Paulhus (2014) developed the 27-item Short Dark Triad scale (SD3), and nine of its items measure narcissism. Do and Dadvari (2017) used these nine items in the SD3 to measure narcissism traits among college students in Taiwan, and found that narcissism was significantly correlated with entrepreneurial attitude orientation and entrepreneurial intention.

Besides the several commonly-used measures of narcissism noted above, entrepreneurial researchers have also employed other infrequently-used scales to investigate the relation between narcissism and entrepreneurship. For instance, of the 23 empirical studies identified in this study, Baldegger et al. (2017) used narcissistic admiration and rivalry questionnaire (NARQ), developed by Back et al. (2013), to exam how narcissistic admiration and rivalry influence entrepreneurial intention. Yu et al. (2020) and Al-Ghazali and Afsar (2020) adopted the approach of Resick et al. (2009), asking participants to evaluate the extent to which each of eight adjective words captures their personality toward the narcissistic tendencies, to measure narcissism among participants.

In addition to self-report scales, entrepreneurial researchers have used proxy indicators to measure narcissism. Chatterjee and Hambrick (2007) argued that using self-report scales to measure narcissism levels among senior executives or entrepreneurs may cause low response rates and social desirability bias. Therefore, they extracted certain unobtrusive indicators from annual company reports and other materials as proxy variables for narcissism. This included things like the prominence of the CEO photos in annual reports, their prominence in press releases, the frequency of CEOs' use of first-person singular pronouns in personal interviews, their cash and non-cash compensation divided by the second-highest-paid executives (Chatterjee and Hambrick, 2007, p. 363). Engelen et al. (2016) adopted this method to measure narcissism among CEOs, when examining the impact of CEO's narcissism on the relationship between entrepreneurial orientation and performance. Moreover, of the 23 empirical studies featured in this study, 2 used first-person pronoun usage estimating as the ratio of first-person singular pronouns to total first-person pronouns, based on the study of Raskin and Shaw (1988), to measure the narcissism of crowdfunding entrepreneurs (e.g., Bollaert et al., 2019; Butticè and Rovelli, 2020).

#### Relation Between Narcissism and Entrepreneurship Research

**Figure 2:**
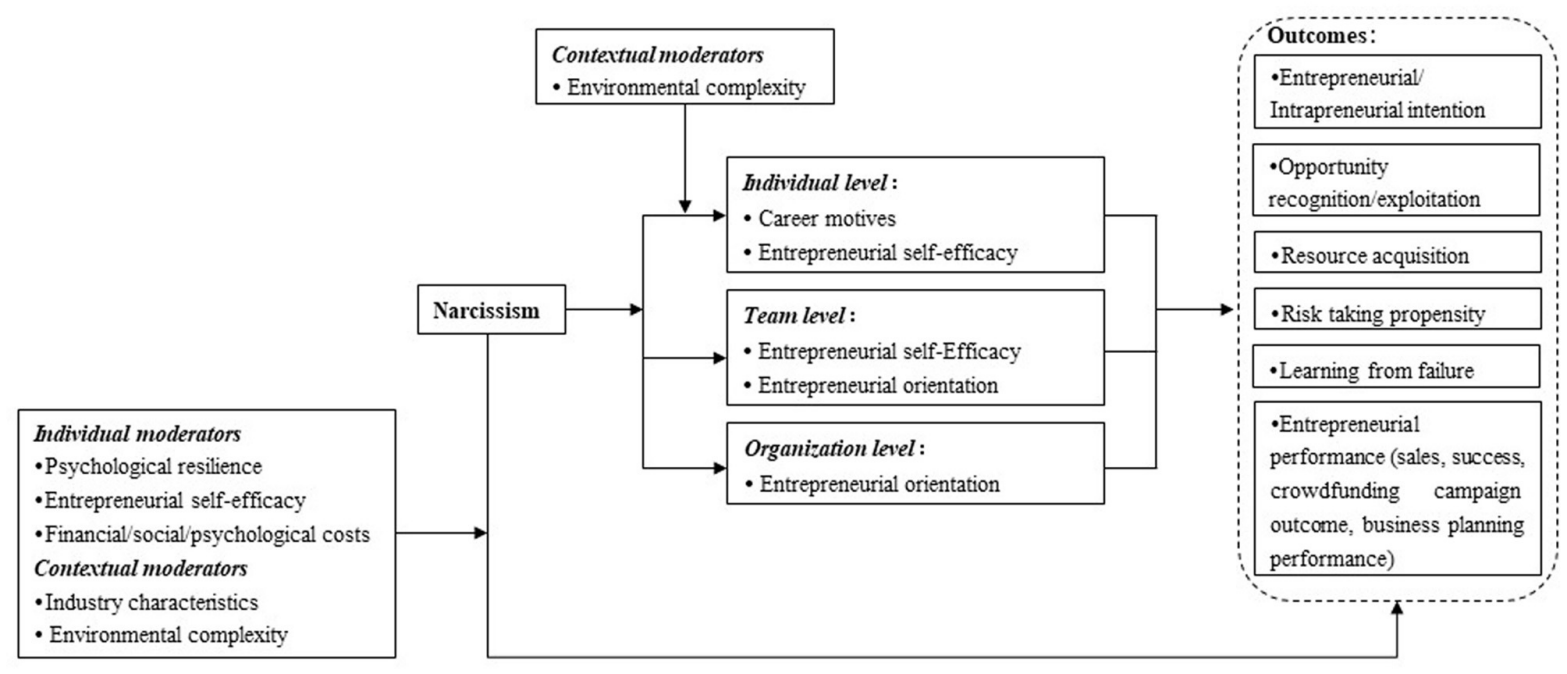
Framework summarizing extent research on narcissism and entrepreneurship.

The findings are presented along the six primary entrepreneurial variables, including entrepreneurial intention, opportunity recognition, resource acquisition, risk-taking, learning from failure, and performance. Figure 2 provides a visual mapping of the review findings, reflecting how narcissism effects entrepreneurial variables.

##### Narcissism and Entrepreneurial Intention

Entrepreneurial intention, a popular and a most studied topic in existing entrepreneurship research, refers to a potential entrepreneurs' subjective stance on whether they should engage in entrepreneurial activities. The concept encompasses the individual's psychological willingness to devote attention and energy to the pursuit of entrepreneurial goals (Thompson, 2009), and is a strong predictor of individuals' engagement in entrepreneurial activities (Yu et al., 2020).

Existing research on narcissism and entrepreneurial intention has mainly focused on two themes. The first centers the main effects that exist between narcissism and entrepreneurial intention, usually answering general questions, such as, “why entrepreneurship attracts narcissists” and “why narcissists have stronger entrepreneurial intentions” from a perspective of fit (e.g., P-E fit theory, career choice theory, and life history theory). For instance, using P-E fit theory and career choice theory, Mathieu and St-Jean (2013) confirmed that narcissistic individuals would choose entrepreneurship because it fits their values and personal characteristics. Their research also demonstrated that entrepreneurs have higher levels of narcissism than non-entrepreneurs (Mathieu and St-Jean, 2013). By adopting the perspective of life history theory, Do and Dadvari (2017) and Hmieleski and Lerner (2016) posited that on the one hand, narcissists value short-term gains and crave the persistent admiration and attention of others, while on the other hand, entrepreneurship enables the attraction of greater social attention, which can satisfy the narcissist's need for attention from others and maintain their sense of superiority. As a result, narcissists tend to employ fast life strategies to regulate their actions and are more willing to experiment with starting new businesses.

The second theme explores the mediation and moderation mechanism between narcissism and entrepreneurial intention. On one hand, the studies examined factors that moderate the relationship between the former and the latter. For example, using the expectancy theory, Tucker et al. (2017) found that entrepreneurial self-efficacy could weaken the positive correlation between employee narcissism and intrapreneurial intentions. Specifically, when entrepreneurial self-efficacy was high, with the increasing level of narcissism, employee's intrapreneurial intention decreased significantly (Tucker et al., 2017). Similarly, based on social exchange theory and social cognitive theory, Wu et al. (2019a) found that psychological resilience also weakened the positive correlation between narcissism and entrepreneurial intention. It must be noted that the differences in culture and entrepreneurial environment may affect the consistency of results related to narcissism and entrepreneurial intention. For instance, Wu et al. (2019b) investigated a sample of MBA students from a Chinese university and found a different relationship between narcissism and entrepreneurial intention compared to conclusions in Western studies. Specifically, they found that narcissism not only was negatively related to entrepreneurial intention, but also had a U-shaped relationship with entrepreneurial intention (Wu et al., 2019b).

On the other hand, the studies also explored the mediation mechanisms between narcissism and entrepreneurial intention. A few studies have shown that there were two mediation mechanisms (cognitive and motivational mechanisms) through which narcissism affected entrepreneurial intention. In terms of cognitive mechanism, researchers found that self-efficacy played an important mediating role between narcissism and entrepreneurial intention (Stöckmann et al., 2015; Wu et al., 2019b; Al-Ghazali and Afsar, 2020). Notably, their research revealed that the indirect relation between narcissism and entrepreneurial intention might be moderated by contextual factors. In particular, using a sample from China, Wu et al. (2019b) found that narcissistic individuals had a lower entrepreneurial self-efficacy which in turn reduced their entrepreneurial intention. On the contrary, Al-Ghazali and Afsar's (2020) research showed that narcissism had a positive indirect effect on entrepreneurial intention via entrepreneurial self-efficacy in Saudi Arabian context. Furthermore, they found that this indirect effect was significant when environmental complexity was high, whereas the indirect effect was insignificant at a low level of environmental complexity.

Regarding motivational mechanism, in addition to the direct effect and cognitive mechanism existing between narcissism and entrepreneurial intention, recent research demonstrated that narcissism also affects entrepreneurial intention indirectly through career motives (Baldegger et al., 2017). For instance, based on the action-characteristics model, Baldegger et al. (2017) studied the relationship between the personality characteristics of dual narcissism (narcissistic admiration and narcissistic rivalry) and entrepreneurial intention. They found that narcissistic admiration affects entrepreneurial intention through the authority and self-realization aspects of career motivation. Meanwhile, narcissistic rivalry affects entrepreneurial intention through the challenge aspects of career motivation (Baldegger et al., 2017).

##### Narcissism and Opportunity Recognition

Entrepreneurial opportunities are subjective, they depend on the entrepreneur's abilities, as well as their beliefs concerning the ability to create value, meet market demand and harvest from competitive imperfections they perceive (Navis and Ozbek, 2016, p. 115). Although entrepreneurial opportunity recognition is an important research topic deserving our research effort (Shane and Venkataraman, 2000), only few studies have explored the relationship between narcissism and opportunity recognition. Moreover, they have primarily focused on reasoning and theory building (e.g., Navis and Ozbek, 2016, 2017; Tucker et al., 2016; Ahsan, 2017), with researchers employing logical deduction to analyze why narcissism affects opportunity recognition.

Previous research suggests that narcissists' desire for self-enhancement, need for praise and admiration from others, and concern for self might prevent them from using the human capital of those around them in the opportunity discovery process, which has a detrimental effect on discovering opportunity (Galvin et al., 2015; Tucker et al., 2016). There were mainly two reasons for this. First, narcissistic individuals consider they are superior to others and have a sense of entitlement, so, narcissist is unwilling to hear from others unless the voice from others can valid self-enhancing nature of narcissist (Grijalva and Harms, 2014; Tucker et al., 2016). Another reason is that narcissistic individuals are resistant to any criticism directed at them or opportunity discovered in order to protect and enhance their grandiose ego, even though the criticism is beneficial for refining the opportunity (Tucker et al., 2016).

##### Narcissism and Resource Acquisition

The effective acquisition and optimal integration of resources are key aspects of the entrepreneurial process. Researchers have used the social exchange theory to argue that narcissistic individuals more successfully obtain early stakeholder (e.g., team members and interviewers) buy-in, due to their positive characteristics (e.g., confidence, extroversion, and passion) (Campbell et al., 2011; Hmieleski and Lerner, 2016). Furthermore, when facing risky environments with high levels of uncertainty, narcissistic entrepreneurs can rely on these positive traits to convey psychological security to their team members by encouraging them to transform uncertainty into opportunity. This will strengthen the team members' trust and support for the narcissistic entrepreneur, which, in turn, facilitates the latter's ability to exploit high-quality social relationships characterized by trust and commitment to acquire the team members' resources (Sundermeier et al., 2020). However, this advantage is not sustainable, as the interactions evolve over time. The positive traits initially exhibited by narcissistic entrepreneurs will be gradually overtaken by negative ones (e.g., selfishness, exploitation, and manipulation), which will diminish stakeholder trust, thereby preventing narcissistic entrepreneurs from establishing long-term benefit exchange (Hmieleski and Lerner, 2016; Wiklund et al., 2018).

In addition to the above analytical and reasoning studies, recent researchers used crowdfunding data to study the impact of entrepreneurs' narcissism on the crowdfunding outcomes of their teams. Studies by Bollaert et al. (2019) and Butticè and Rovelli (2020) indicated that narcissistic entrepreneurs were less likely succeed in resource acquisition (crowdfunding success). Specifically, Bollaert et al. (2019) found that narcissistic entrepreneurs preferred to set a lower funding goal and longer campaign duration than non-narcissistic ones, which attracts fewer backers and less funds. Butticè and Rovelli (2020, p. 4) provided further support that entrepreneur's narcissism was significantly and negatively correlated with the crowdfunding success, the relation between them was even stronger in the industries art, design, film, food, journalism, and theater. Although their research shed light on the consequences of entrepreneur's narcissism, our comprehensive analysis reveals that previous studies have analyzed or empirically examined the impact of entrepreneur's individual narcissism on resource acquisition, few examines whether the potential impacts differ at the entrepreneurial team level. Research in this area needs to be strengthened in the future.

##### Narcissism and Risk-Taking

Most narcissistic entrepreneurs are extremely eager to succeed. The radical, bold, decisive, and entirely self-confident nature of their personalities create a greater willingness to take risks (Campbell et al., 2004b; Foster et al., 2009). There are two main reasons for this. From a cognitive perspective, narcissists have greater self-esteem and higher self-evaluation (Campbell et al., 2011). Their inflated self-perception leads them to overestimate their actual skills and abilities, while underestimating the challenges and difficulties of the entrepreneurial activities (Navis and Ozbek, 2016). Furthermore, this cognitive tendency causes them to be more optimistic about project returns and maintain a higher risk tolerance. Consequently, they are more likely to be attracted to high-risk, high-reward projects (Gerstner et al., 2013). From a motivational perspective, narcissists constantly seek admiration and attention from others, pursuing a sense of superiority and power which gives them a higher risk tolerance (Yu et al., 2020). Thus, although entrepreneurship is inherently risky, narcissists still have a greater willingness to engage in it, to gain the social attention and power that entrepreneurship brings (Mathieu and St-Jean, 2013; Hmieleski and Lerner, 2016).

##### Narcissism and Learning from Failure

Researchers have long acknowledged that failure promotes learning (Shepherd, 2003; Cope, 2011). Learning from failure can be beneficial to entrepreneurs, such as improving their reflexive ability and resilience (Cope, 2011), as well as the quality of their decision-making (McGrath, 1964). Hence, many previous researches about learning from failure have focused on improving the effectiveness of this learning. However, recent studies have found that, to protect their fragile, high self-esteem, narcissistic entrepreneurs are often reluctant to admit failure—let alone learn from it (Navis and Ozbek, 2016; Liu et al., 2019). Liu et al. (2019) surveyed 180 entrepreneurs who had failure experience in the past three years, and found that entrepreneurs' narcissism is not conducive to their learning from entrepreneurial failures. This is especially so when they have incurred great social (compared to financial and psychological) costs, which further contribute to the lack of motivation to learn from failure (Liu et al., 2019).

According to literature we identified, there are two main reasons why narcissism prevents entrepreneurs from learning from failure. First, narcissists have an inflated positive view of self, a motivation to maintain and enhance their positive self-view, and a strong sense of superiority (Campbell et al., 2011). When faced with failure, highly narcissistic entrepreneurs attempt to maintain their grandiose positive self-view and high but fragile self-esteem by selectively attending to information and cues that confirm their original visions (Navis and Ozbek, 2016). Moreover, they may not believe that information from others could be valuable. The above-described cognitive biases can prevent them from attending to and understanding the causes of failure, and thus learning from it. Second, narcissistic entrepreneurs tend to adopt external-attribution ego-defensive strategies to deny or excuse their own failures (Judge et al., 2006). This tendency also can prevent critical reflection and is not conducive to learning from failure (Cope, 2011). As Kets de Vries and Miller (1985) suggest, the narcissist's natural sense of superiority and arrogance can make them reluctant to admit failure or promptly discontinue unsuccessful actions.

##### Narcissism and Entrepreneurial Performance

Existing research mainly examines the impact of narcissism using two types of indicators for entrepreneurial performance. One focuses on financial indicators (e.g., sales revenue and its fluctuations, shareholder value, gross profit, and net profit). Research in this area primarily examines the role of entrepreneurially-oriented strategy in the relationship between narcissistic CEOs and firm performance (from the perspective of corporate strategy). For example, Wales et al. (2013) found that the perceptions and preferences of narcissistic CEOs motivate them to implement high-risk, entrepreneurially-oriented company strategies that resulted in significant sales revenue fluctuations. Others found that, in a fragmented and stable market, a narcissistic CEO weakens the positive correlation between a firm's entrepreneurial orientation and shareholder value (Engelen et al., 2016). It is worth noting, however, that Kraus et al.'s (2018) study on small and medium-sized European enterprises showed that narcissism among top executives did not moderate the positive relationship between entrepreneurial orientation and overall performance.

The other type of indicator measures the effect of narcissism on entrepreneurial performance by focusing on the innovation and growth of startups. For example, Leonelli et al. (2019) found an inverted U-shaped relationship between entrepreneurial narcissism and startup innovation at the individual level. Moreover, this curvilinear relationship was attenuated in dynamic markets and unaffected in concentrated markets. Stöckmann et al. (2015) examined the pathways by which narcissism influences the business planning performance of entrepreneurial teams. They found that the higher the narcissistic team member's narcissism, the more likely it is that the narcissistic team member will use his/her oratory skills to positively influence the team's aggregated level of entrepreneurial self-efficacy. This results in team members maintaining positive attitudes toward entrepreneurship, which can ultimately affect the team's entrepreneurial planning performance. Kollmann et al. (2019) verified that the level of narcissism in entrepreneurial teams strengthened the positive correlation between task conflicts and business planning performance. Besides, they also found that the moderating role played by the team's level of narcissism is stronger when team member's actual and perceived entrepreneurial capabilities are higher.

## Discussion and Agenda for Future Research

The aim of this systematic review is to deepen the understanding of and draw more attention to narcissism and entrepreneurship. Upon mapping and integrating existing knowledge, it is clear that narcissism is an important influencing factor for entrepreneurial choice (e.g., intention and entry), entrepreneurial activity (e.g., opportunity recognition, risk-taking, and learning from failure), and entrepreneurial output (e.g., performance). While the results of previous studies were promising in their ability to help researchers understand the relationship between narcissism and entrepreneurship, they do not provide an in-depth investigation on this relationship. For example, the majority of the studies included in the present study used cross-sectional design which limited our ability to explore causality while ignoring the dynamic or non-linear relationship between narcissism and entrepreneurship. Furthermore, they also fail to effectively elucidate the complexities of the relationship between narcissism and entrepreneurship. Hence, based on our systematic analysis, our understanding of the related literature, and identified research gaps, we present an agenda for future research in the remaining sections. This agenda details opportunities to advance the scope of the literature.

### Considering the Time Factor in the Relationship Between Narcissism and Entrepreneurship Variables

Entrepreneurship is a dynamic process that continuously unfolds over time. Accordingly, many entrepreneurial activities and outcomes (such as the assessment of entrepreneurial opportunity and the interactions within entrepreneurial teams and entrepreneurial performance) evolve or take on different characteristics over time, too. Indeed, time affects entrepreneurship at the micro (individual), meso (corporate), and macro (culture etc.) levels. Centering time (and its impact on individual entrepreneurs, entrepreneurial ventures, and the entrepreneurial environment) may lead researchers to a better understanding of entrepreneurship (Levesque and Stephan, 2020, p. 164). For this reason, scholars have called for much-needed research on the significant role of time in various entrepreneurial activities and phenomena (McMullen and Dimov, 2013; Grijalva and Harms, 2014; Levesque and Stephan, 2020). However, a vast majority of researchers who are concerned with the relationship in question used cross-sectional data to conduct their studies (e.g., Hmieleski and Lerner, 2016; Tucker et al., 2017, Baldegger et al., 2017; Jackson, 2018 also see Table 1), and overlooked the importance of time. This is not conducive to researchers' exploration of the causal or dynamic relationship between variables, nor does it help transform the researchers' prior perspectives (e.g., studying entrepreneurship as an act, McMullen and Dimov, 2013) in preparation for in-depth analysis of the entrepreneurial process.

In fact, a few studies imply a strong need to consider time when exploring: (1) the relationship between narcissism and entrepreneurship variables, and (2) the impact of narcissism on the entrepreneurial process. For example, based on the analysis of previous studies, Tucker et al. (2016) revealed that narcissism has different roles in various entrepreneurial stages. In the opportunity recognition stage, narcissists are unlikely to effectively use human capital of others. In the opportunity evaluation stage, they use the capital of others to meet their own gain. In the opportunity exploitation stage, while narcissists take bold action to exploit opportunities, their behavior is not based on effectively leveraging the capabilities of those around them. Mathieu and St-Jean (2013) and Wu et al. (2019b) found that while narcissism positively affects entrepreneurial intention, it does not necessarily have a positive impact on success or long-term performance. This is because of their previously-mentioned focus on short-term gains at the expense of long-term ones.

Organizational behavior research has long considered the role of time. This approach provides a lens through which future research may explore the relationship between narcissism and entrepreneurship. The findings of existing literature suggest that researchers can use the following perspectives to incorporate time: temporal variables, causalities in temporal precedence, temporal context, and temporal patterns (Sonnentag, 2012; Vantilborgh et al., 2018; Levesque and Stephan, 2020). More specifically, temporal variables incorporate the element of time at the variable level, and time is often an important component of such variables (Sonnentag, 2012; Vantilborgh et al., 2018). Examples within this category include time-consciousness (Levesque and Stephan, 2020), time management (Levesque and Stephan, 2020), temporal leadership (Chen and Nadkarni, 2017), and time urgency (Chen and Nadkarni, 2017). The study of temporal variables is a direct way to incorporate time into our research on the relationship between narcissism and entrepreneurship. By taking temporal variables into account, researchers can examine the underlying relationships or mechanisms between entrepreneurs' narcissism and their time-perception, time-consciousness, and entrepreneurial (long- and short-term) decision-making. They can also explore how narcissistic entrepreneurs manage their time in different entrepreneurship process and activities.

A second way to account for time is to use longitudinal designs or latent change score modeling to investigate causalities in the temporal precedence of narcissism and entrepreneurship-related variables. Previous research shows that narcissistic traits are not static, and narcissism levels may be affected by social factors (competitive social environment, use of social media, etc.; Thomaes et al., 2018). Entrepreneurial success may further reinforce the narcissism of entrepreneurs who have worked hard to achieve their success (Grijalva and Harms, 2014). Therefore, with the help of the latent change score model (McArdle, 2009), researchers can investigate whether there is a reciprocal relationship between changes in narcissism and changes in entrepreneurial performance. They can also investigate the influencing factors of this relationship: for example, (1) whether narcissistic entrepreneurs are successful, and under what circumstances, and (2) how and when entrepreneurial success influences (strengthens or weakens) the entrepreneur's level of narcissism.

A third approach is to consider time a contextual factor or moderating variable in our study of the relationship between narcissism and entrepreneurship-related variables. This enables researchers to gain greater insight into entrepreneurial activities or phenomena at a given time or during a particular period. Tucker et al.'s (2016) analysis on narcissistic entrepreneurs' effective use of human capital felicitously illustrates the role of time as a contextual factor. In accordance with the different stages of entrepreneurship and industry development, future research can investigate different relationships which may exist between narcissism and entrepreneurial team processes (e.g., team conflicts), decision-making, and performance. Researchers can also study the differences and characteristics of time management and risk attitudes exhibited by narcissistic entrepreneurs at different stages of their entrepreneurship.

The final approach is to use temporal patterns: a more complex way to explore the role of time. This approach reflects the ways in which variables—or the relationships between them—change over time. It also provides information about the evolution of dynamic phenomena over time (Vantilborgh et al., 2018). In the existing literature, researchers have mostly studied temporal patterns with respect to the trajectories, trends, stability, or periodicity of the variables of interest—or the relationships between them. For example, Uitdewilligen et al. (2018) used pattern detection algorithm software to track: (1) the emergent performative patterns of task-oriented behaviors among team members over time, and (2) the dynamic relationship of such action patterns in relation to overall team effectiveness. By incorporating time, researchers can uncover the dynamic evolution of narcissistic entrepreneurs' entrepreneurial activities and the relationship between narcissism and entrepreneurship-related variables over time. In the future, targeted research may center the trajectories, trends, stability, or periodicity of variables, such as entrepreneurial performance and the passion of narcissistic entrepreneurs. The relationship between narcissism and entrepreneurial performance may undergo dynamic changes over time. These shifts may be reflected in the variations of average values, the correlations between performance dimensions, or even the stability or periodicity exhibited by performance over time.

### Examining the Relationship Between Narcissism and Entrepreneurship at the Team Level

Many startups are created by entrepreneurial teams. The involvement of an effective team is required when coping with the uncertainty, financial pressure, and unique challenges faced by startups. Moreover, this involvement is needed to take advantage of opportunities and make key decisions (West, 2007; Schjoedt et al., 2013; De Mol et al., 2015). Research shows that the formation, composition, and functioning of entrepreneurial teams may profoundly impact entrepreneurial outcomes, such as the growth of new ventures. For example, the meta-analysis of Jin et al. (2017) found that the characteristics of entrepreneurial teams (e.g., team size, average team member experience, heterogeneity related to age, gender, and work experience) have a significantly positive impact on the performance of startups. The results of Kollmann et al. (2019) illustrate that team task conflict can effectively improve the quality of business plans in student teams. De Mol et al. (2015) analyzed 44 studies on entrepreneurial team cognition and demonstrated that it could promote information processing, opportunity recognition, decision-making efficiency, and team learning. It could also improve entrepreneurial performance and affect the recruitment practices through networks.

Despite the importance of entrepreneurial teams, our in-depth analysis of previous literature reveals that the vast majority of studies have only verified the crucial role of narcissism in entrepreneurial intention, recognition, and development of entrepreneurial opportunities, and other entrepreneurial activities at the individual level (e.g., Mathieu and St-Jean, 2013; Do and Dadvari, 2017; Bollaert et al., 2019; Wu et al., 2019b). In contrast, only a few researchers have explored whether narcissism may have different potential effects on activities (or phenomena) at the level of entrepreneurial teams. In fact, a handful of studies have already obtained preliminary findings suggesting both the number of narcissistic members in a team and their average narcissism level can significantly affect its process and outcomes. For example, Kollmann et al. (2019) found that narcissism can shape intra-team interactions and enhance the exchange of different views and information concerning team tasks. Furthermore, the level of team narcissism can reinforce the positive relationship between team task conflict and the quality of business plans. Goncalo et al. (2010) showed that having more narcissistic team members is not necessarily beneficial. This is due to an inverted U-shaped relationship between the number of narcissistic members and systematic thinking of the collective while problem solving. A similar inverted U-shaped relationship was also identified between the average level of team narcissism and innovation outcomes. Their findings suggest that conclusions drawn from doing so may be more excited and remarkable that those at the individual level.

To investigate the former, researchers can first study the ways in which narcissistic entrepreneurs form their own startup teams. Most research on the relationship between the characteristics and composition of entrepreneurial teams and entrepreneurial performance has a basic premise that entrepreneurial teams already exist (e.g., Klotz et al., 2014; Jin et al., 2017). These studies, which reflect the “input-output” or “input-process-output” models, tend to overlook some critical issues, such as the way entrepreneurial teams are formed (e.g., formation sequence, timing, dynamics) and the factors that influence this formation (e.g., co-founder selection strategy, Lazar et al., 2020). Here, are some topics worthy of further study: (1) What kind of team members would a narcissistic entrepreneur choose: narcissistic or complementary? (2) By what means do narcissistic entrepreneurs (e.g., interpersonal-attraction strategy or resource-seeking strategy), as in Lazar et al. (2020) select their team members? (3) how effective are the team-building strategies of narcissistic entrepreneurs? (4) What is the dynamic process of forming a startup team for narcissistic entrepreneurs (e.g., team members joining and leaving the team)?, and (5) What are the contextual factors that can influence a narcissistic entrepreneur's formation of a startup team and its dynamic process?

Second, researchers can examine how narcissistic team leaders affect the processes and performance of the entrepreneurial team. Studies in the field of leadership and teamwork show that leaders can have a crucial impact in these areas. Indeed, leaders may directly or indirectly influence numerous team-level variables, including cohesion (e.g., Chiniara and Bentein, 2018), cooperation (e.g., Zhang et al., 2011), conflict (e.g., Kotlyar et al., 2011; Schraub et al., 2014), efficacy (e.g., Srivastava et al., 2006), innovation (e.g., Chen, 2007; Yin et al., 2020), and performance (e.g., Zhang et al., 2011; Owens and Hekman, 2016). Existing team theories provided a wealth of literature which aided our understanding of entrepreneurial teams. Yet, given the unique nature of entrepreneurial teams and the environments they face (e.g., endogeneity, Lazar et al., 2020); vulnerable social situations, Klotz et al., 2014), we believe it necessary to enhance our understanding of the ways in which those led by narcissistic leaders can maximize their operation and efficacy. Future studies may consider the dynamic relationships of narcissistic team leaders and entrepreneurial team processes (e.g., team process and cognitive-emotional processes) in relation to opportunity recognition, resource acquisition, learning from failure, and entrepreneurial performance. Moreover, they may consider whether these relationships are influenced by contextual factors (e.g., uncertainty and industry).

Third, in addition to the topics above, it is equally important to examine the mechanisms among interaction of multiple narcissistic team members, team processes, and entrepreneurial outcomes. Prior research indicates that narcissism can positively influence an individual's entrepreneurial intention (Mathieu and St-Jean, 2013; Hmieleski and Lerner, 2016; Tucker et al., 2017), encourage discussions about tasks among team members, and reinforce the positive relationship between team task conflict and team performance (Kollmann et al., 2019). However, some studies have suggested that it is not necessarily better to have more narcissistic members in a team (Goncalo et al., 2010). Hence, the questions that arise are: (1) What is the proper number (or percentage) of narcissistic members needed in a startup team for it to facilitate team functioning? and (2) When and how does narcissistic heterogeneity and the average level of narcissism among entrepreneurial team members influence entrepreneurial outcomes through team processes? Researchers can refer to the insights offered by the framework of Klotz et al. (2014) to study how the number of narcissistic members, narcissistic heterogeneity, and average level of narcissism influence entrepreneurial outcomes (sales growth, profitability, innovation, etc.) through team processes (team member changes, team conflicts, team planning, etc.), and cognitive-emotional states (team cohesion, team self-efficacy, etc.). Researchers can also develop and test conditional indirect models that determine when and how the number of team narcissistic members, narcissistic heterogeneity, and average level of narcissism affect the performance of entrepreneurial teams.

### Exploring the Relationship Between Narcissism and Entrepreneurial Ethics

In recent years, the relationship between entrepreneurship and ethics has become an increasingly popular topic among researchers and in the media (Griffith, 2017; Ahsan, 2018; Vallaster et al., 2019). Entrepreneurship not only drives innovation, provides more job opportunities, and promotes economic development (Hannafey, 2003; Scott et al., 2014), it also helps entrepreneurs achieve their goals (Baron et al., 2015; Hmieleski and Lerner, 2016). However, any entrepreneurial activity undertaken may be inextricably intertwined with ethics. Entrepreneurs inevitably face many ethics-related issues, such as ethical decision-making dilemmas that directly affect company performance, disruptive innovation, and enforcement of ethical standards, honest communication, and truthful disclosure (Morris et al., 2002; Harris et al., 2009; Vallaster et al., 2019). Research has shown that among the many factors that influence entrepreneurs' unethical motivations and behaviors, unique personality traits can influence their reasoning, attitudes, behavioral tendencies, and behavior in relation to ethical issues (Hannafey, 2003). Narcissism is one of the traits that has not only been shown to be pertinent to entrepreneurship (Mathieu and St-Jean, 2013; Wu et al., 2019a), but also intricately linked to unethical motives and behavior (Grijalva and Newman, 2015; Campbell and Siedor, 2016; Hmieleski and Lerner, 2016). However, this trait has not received much attention from researchers in the field of entrepreneurship ethics. As the analysis in section Results and Findings demonstrates, literature on the relationship between narcissism and entrepreneurship rarely addresses ethical issues (except Yu et al., 2020). Similarly, research on the relationship between narcissism and unethical motivations and behavior shows that narcissistic individuals have strong self-serving motivations. This is accompanied by a lack of empathy (Campbell et al., 2011) and ethical sensitivity (Roberts, 2001). Furthermore, narcissism can increase an individual's counterproductive behavior (Grijalva and Newman, 2015), predict higher levels of dishonesty (Campbell and Siedor, 2016), and reduce ethical leadership behavior (Hoffman et al., 2013). Notably, however, these studies do not specifically target entrepreneurial contexts. The relationship between narcissism and entrepreneurship has received considerable attention from researchers, as has that of narcissism and non-ethical motivations and behaviors. Still, the intersection of narcissism, entrepreneurship, and ethics has not been adequately explored. Hence, exploring the ethical decision-making mechanisms of narcissistic entrepreneurs (and how they influence the ethical norms and behaviors of startups) is useful for integrating these three domains. It is also useful for expanding the theoretical and empirical study of entrepreneurial ethics. Thus, it is necessary for future research to explore ways to integrate the literature in these fields, to guide further research.

According to Morris et al. (2002, p. 331), the intersection of entrepreneurship and ethics involves two major themes, namely: ethics in entrepreneurial contexts and the ethical contexts of entrepreneurship. The former is concerned with some of the unique ethical challenges faced in entrepreneurial contexts, and the entrepreneurs' ethical judgments and behaviors in response; for example, the relationship between financial/resource-related pressures and entrepreneurial decisions. The latter focuses on the ethical climate established in startups, such as the mechanisms put in place by entrepreneurs to ensure compliance with ethical standards.

As summarized by Morris et al. (2002), these two overarching themes offer valuable ideas for investigating the intersection of narcissism, entrepreneurship, and ethics. This avails some potential avenues future researchers may wish to consider. First, researchers can study the factors that underpin the moral and immoral decisions made by narcissistic entrepreneurs. Narcissistic individuals have strong self-serving motives and a tendency to exploit others for personal gain. They are often self-centered, lack concern and empathy for others, lack ethical sensitivity, and rarely consider the impact of their decisions on others (Roberts, 2001; Campbell et al., 2005, 2011). It is precisely because of these characteristics that, cognitively, narcissistic individuals cannot recognize moral problems, initiate moral disengagement processes, and often make moral judgments that deviate from ethical standards (Cooper and Pullig, 2013; Jones et al., 2017; Erzi, 2020); while emotionally, they are often unable to fully experience ethical emotions such as guilt (Campbell et al., 2004a; Liu et al., 2019; Schröder-Abé and Fatfouta, 2019). Notably, both these cognitive and emotional components can trigger unethical behavior (Jones et al., 2017; Schröder-Abé and Fatfouta, 2019).

The above analysis suggests that both the cognition and moral emotions of narcissistic entrepreneurs may have a noteworthy impact on their ethical decision-making. Moreover, it is clear that two types of mechanisms (i.e., cognitive and emotional) are involved in the relationship between entrepreneurial narcissism and ethical behaviors. Future research could identify contexts and processes that may lead narcissistic entrepreneurs to make better moral decisions. Studies may also explore the factors that can arouse their moral emotions, in order to sensitize them to ethical issues, restrict their moral disengagement, and bolster their moral judgments, which can serve as a basis to encourage actions that conform to ethical standards. In addition, future research could also investigate whether and how arousing the moral emotions of narcissistic entrepreneurs influences their cognitive processes in relation to ethical decision-making (e.g., rules-based or cost/benefit-based cognitive reasoning, Tipu, 2015), or *vice versa*.

Second, researchers can examine how narcissistic entrepreneurs influence the ethical norms and climate of startups. The body of research examining the influences of moral climate (see Newman et al., 2017) has been growing. However, since Morris et al.'s (2002) initial study on the formation of ethical climate in startups, research on the antecedents, and formation mechanisms of ethical climate in startups has been scarce and slow-paced. This leaves researchers with important, unanswered questions, for instance: (1) How are the ethical norms and ethical climate formed in startups founded by narcissistic entrepreneurs? (2) How do they evolve with the entrepreneurial process? (3) How sustainable are they? (4) What effects do the heterogeneity of ethical sensitivity, moral values, or heterogeneity of standards among entrepreneurial team members have on the formation of ethical norms and ethical climate in startups? (5) How do external factors (national culture, intense market competition, resource pressures, etc.) affect the formation and changes of ethical norms and ethical climate in startups? and (6) How do narcissistic and non-narcissistic entrepreneurs differ in their influence over the ethical norms and ethical climate of startups? These are all important prompts worth investigating.

Besides the two aforementioned directions, future research could explore the ways in which narcissistic entrepreneurs influence the social responsibility of startups (Tiba et al., 2019; Vallaster et al., 2019). Although previous studies on social entrepreneurship have centered on social responsibility, these studies tend to focus on non-profit organizations, overlooking social responsibility in for-profit commercial startups (Tiba et al., 2019). Narcissistic individuals have strong self-serving and self-enhancement motivations, they crave attention, recognition, and admiration from others, and they focus on self-interest and short-term benefits (Campbell et al., 2011; Hmieleski and Lerner, 2016). Thus, in the face of challenges (such as resource constraints and competitive pressures) narcissistic entrepreneurs may take on social responsibility work to gain attention and create a positive image. Indeed, this would not necessarily be due to a sincere desire to fulfill their social responsibilities (Roberts, 2001; Hellmich and Hellmich, 2019). Future research can systematically investigate what factors make narcissistic entrepreneurs more socially responsible in new ventures; how narcissistic entrepreneurs influence the startup's practices in the three areas of the “3Ps” social responsibility model (Elkington, 1998; Tiba et al., 2019); as well as whether and how startup performance in the “3Ps” may affect the subsequent decision-making of narcissistic entrepreneurs (investment in social responsibility, ethical decision-making, corporate R&D investment, etc.).

### Investigating the Interaction Effects That Narcissism and Bright Traits or Other Dark Traits on Entrepreneurial Activities and Outcomes

Most of the existing scholarship has focused on the simple relationship between personality and entrepreneurial activities and outcomes. However, a growing body of research suggests that all personality traits—bright (e.g., optimism) or dark (e.g., narcissism)—are likely to have upsides and downsides (Smith et al., 2018, p. 193). Thus, the relationship between personality traits and entrepreneurial activities and outcomes may be far more complex than previously observed (DeNisi, 2015; Klotz and Neubaum, 2016; Miller, 2016). One way to address this complex issue is to examine how the interactions between entrepreneurs' personalities, and the interactions between investor-entrepreneur personalities, affect entrepreneurial activities and outcomes. Researchers have called for greater understanding of the interaction effects of entrepreneurial personalities and of investor-entrepreneur personalities (e.g., Murnieks et al., 2011; Klotz and Neubaum, 2016; Smith et al., 2018; Levesque and Stephan, 2020). Yet, limited progress has been made in these areas compared to others (e.g., how personality traits independently, as opposed to interactively, predict entrepreneurial activities or outcomes). Making use of this research opportunity will lead to a deeper and more systematic understanding of how interactions between entrepreneurial personalities, and the interactions between investor-entrepreneur personalities, affect entrepreneurial activities and outcomes.

Indeed, previous research indicates that it is not without foundation to investigate how the interaction of narcissism and other personality traits affects outcomes in the field of entrepreneurship. Paradox theory reveals both the coexistence of different or even opposing personality traits (dark and bright) and the impact of their interaction (Lewis, 2000; Smith and Lewis, 2011). Thus, it provides a theoretical basis for researchers to study the interaction of narcissism with other personality traits. For example, Owens et al. (2015) examined how humility interacts with narcissism to impact leadership effectiveness. They found that humility can make narcissistic leaders more effective because it involves the recognition of other people's strengths and contributions, acknowledgment of one's own shortcomings, and acceptance of other people's opinions and feedback. This can counteract or neutralize the negative effects of narcissism. Similarly, Zhang et al. (2017) studied the impact of CEO narcissism and humility on the firm innovation culture and performance. They found that CEOs who are both narcissistic and humble not only improve employees' ratings of their charisma, they enhance the firm's innovation culture and performance.

In the field of narcissism and entrepreneurship, we found that only Wu et al. (2019a) and Navis and Ozbek (2016) conducted research on the interplay between narcissism and bright traits. Wu et al. (2019a) explored the effect of the interaction of dark and bright personality traits on sustainable entrepreneurial orientation, and the results showed that psychological resilience can weaken the relationship between narcissism and sustainable entrepreneurial orientation. Navis and Ozbek (2016) analyzed the effects of the interaction between narcissism and overconfidence. They found that highly narcissistic and overconfident entrepreneurs are more likely to start businesses in novel environments than in familiar environments. These studies provide valuable references for future researchers to conduct in-depth studies. By drawing on and expanding these studies, researchers can conduct further research to examine how bright traits (e.g., humility and self-awareness) affect the relationship between narcissism and entrepreneurial activities and outcomes. Moreover, they can determine whether different bright traits may play different roles. For example, humility may inhibit the negative effects of narcissism on entrepreneurial learning, long-term entrepreneurial development orientation, and R&D investment, whereas self-awareness may inhibit unethical behavior by narcissistic entrepreneurs.

In addition, future researchers could study the impact that the interaction between investors' personalities and entrepreneurs' narcissistic personality may have on entrepreneurial activities and outcomes. Investors' influence on entrepreneurial activities and outcomes often exists during venture financing and the subsequent operating phase (Drover et al., 2017). Prior research yielded promising results in terms of investors' investment decisions and strategies (e.g., Burchardt et al., 2016; Davis et al., 2017), startups' financing methods and outcomes (e.g., Anglin et al., 2018; Bollaert et al., 2019; Butticè and Rovelli, 2020), and startups' development and performance after receiving financing (e.g., Croce et al., 2013; Guerini and Quas, 2016). Yet, few studies address the impact of the investor-entrepreneur dyad on entrepreneurial activities and outcomes. Future research can investigate how investors' bright (e.g., confident) or dark (e.g., narcissistic) personality traits influence narcissistic entrepreneurs' entrepreneurial activities and outcomes (e.g., financing, growth). The findings would make a valuable contribution to new perspectives on how the investor-entrepreneur personality fit affects entrepreneurial activity and outcomes, thereby enriching and extending the research on the investor-entrepreneur dyad.

While the present review demonstrates the characteristics and nature of the relations between narcissism and entrepreneurship and provides above directions worthy of future research, there are certain limitations that researchers should take into account while using results of present research. First, although a rigorous methodology was employed to conduct the systematic literature review, and the categorizations of six research themes were the result of a systematic step-by-step process, it remains possible that we have missed some studies. For instance, we did not include unpublished articles, therefore we cannot guarantee that all data and important results were covered. Another limitation is that there may be a language bias, because we only selected studies published in English. However, previous research has shown that exclusion of non-English studies rarely impacts the results and conclusion of a review (e.g., Morrison et al., 2012), thus, we believe that language bias has little effect on results and conclusion of the present study. A final limitation concerning the challenges encountered in delving into the above future directions. While each direction affords us a new way of investigating the relation between narcissism and entrepreneurship, they also pose certain challenges for research. For example, challenges moving away from cross-sectional studies and to incorporate time lag and dynamic phenomena changing over time of variables of interest into our research designs. Besides, gathering data to study research questions posed above will also challenge us as a researcher. However, it should be highlighted that we do not provide specific challenges each of them may have, which depends on the specific study of the researcher.

## Conclusion

Based on the literature review, we found that narcissism is an important personality trait that influences entrepreneurial behavior and outcomes. However, despite the call for more research of, and attention to, the role of narcissism in entrepreneurship, studies in this area remain lacking. This article served to review the current state of research on this relationship, in an attempt to strengthen interest in it. Our in-depth analysis of the relationship between narcissism and entrepreneurship was based on effective practices gleaned from previous literature analysis studies. It not only echoes the call for more research, it also provides researchers with a systematic and comprehensive understanding of the findings to date. However, as Stöckmann et al. (2015) noted, unraveling the enigmas of the narcissism-entrepreneurship relationship may not be as simple as it seems, and it may be more complex than implied in previous literature. We hope and believe that, through our in-depth analysis of the existing literature, this study will provide valuable direction of future research. We also expect this study to lay the groundwork for advancing research on personality traits and the integral research of the relationship between narcissism and entrepreneurship, to resolve the mysteries therein.

## Data Availability Statement

The original contributions presented in the study are included in the article/supplementary material, further inquiries can be directed to the corresponding author/s.

## Author Contributions

All authors listed have made a substantial, direct and intellectual contribution to the work, and approved it for publication.

## Conflict of Interest

The authors declare that the research was conducted in the absence of any commercial or financial relationships that could be construed as a potential conflict of interest.
